# A new path for assisted dying in Europe: France's middle way

**DOI:** 10.1016/j.lanepe.2026.101617

**Published:** 2026-02-05

**Authors:** The Lancet Regional Health – Europe

Modern medicine has dramatically extended lifespans, but it has been far less comfortable acknowledging its limits. Nowhere is this tension more visible than in debates on assisted dying, where clinical uncertainty, ethical discomfort, and legal caution collide. Across Europe, this tension has shaped the debate on assisted dying, often leading to polarised positions: on one end, absolute opposition; on the other, more permissive models such as Switzerland, where assisted suicide is facilitated by private organisations and is available even to non-residents.

Amid these extremes, France is forging a ‘middle way’—combining access to assisted dying with a strong commitment to universal palliative care, while rejecting both prohibition and commercialised approaches. At the centre of this shift is the *Projet de loi relatif à l'aide à mourir* (the Bill on End-of-Life Care), which combines access to assisted dying with a sovereign commitment to universal palliative care.

Public support for assisted dying in France is substantial: 92% of citizens and 74% of physicians are in favour of this legalisation. As the bill returns to the National Assembly, the lower chamber, following the Senate's recent push for stricter palliative-first mandates, the debate has centred on ensuring that assisted dying is never a default response to unmet care needs. By anchoring the debate in “laïcité” (secularism) and “fraternité” (brotherhood), France frames a dignified death as a collective guarantee, rather than a private privilege for the few. Safeguards include psychiatric assessment, waiting periods, and mandatory palliative consultations. Central to the proposal is the principle that assisted dying must not serve as a substitute for inadequate palliative care.

These concerns are particularly salient given the current state of France's palliative infrastructure. Despite the investment pledge, a quarter of palliative needs remain unmet, especially in so-called rural ‘medical deserts.’ Beyond the funding gaps, the reform requires a cultural shift where end-of-life care is recognised as a core clinical responsibility rather than a point of medical disengagement. Without this foundation, the ‘middle way’ risks becoming a fallback for systemic neglect rather than a principled ethical alternative.

To support this vision, the French Government has committed €1.1 billion over ten years to expand palliative infrastructure, recognising that meaningful end-of-life choice depends on access to high-quality care. Framing assisted dying within this investment rejects utilitarian narratives that cast it as a cost-saving measure, and instead, affirms that the state's duty of care should be most robust at the end of life. France reframes assisted dying as “*soin d'accompagnement*” (“accompanying care”) integrating it into the care continuum and rejecting the notion that death is where medicine stops caring. To support a holistic approach, the reform introduces community-based “*maisons d'accompagnement*” (accompanying homes) to bridge the gap between sterile hospital wards and potentially isolating homes, and provides long-form clinical geriatric consultations.

Although research suggests that well-regulated legislation can ensure that only those who are genuinely eligible can access assisted dying safely, evidence from other European contexts suggests that requests for assisted dying are shaped by more than access to medical and palliative care alone. Social isolation, limited support, and concerns about becoming a burden seem to play an important role, underscoring that autonomy at the end of life is inseparable from social context.

Furthermore, experience from countries such as the Netherlands and Belgium has complicated the debate, with ongoing discussion about how eligibility for assisted dying has evolved beyond terminal illness to include some chronic non-terminal and psychiatric conditions. These developments have renewed long-standing concerns about the potential for gradual broadening of eligibility once physician-assisted dying is legalised.

By linking assisted dying to equitable care infrastructure, France seeks to reimagine the “right to die” as a “right to be accompanied.” Whether this vision can withstand the realities of implementation—including workforce capacity, regional inequality, and social care provision—will determine whether France's middle way becomes a durable model for Europe, or an aspirational compromise tested by the limits of health and social systems.

